# Amelioration of Scopolamine-Induced Learning and Memory Impairment by *α*-Pinene in C57BL/6 Mice

**DOI:** 10.1155/2017/4926815

**Published:** 2017-11-01

**Authors:** Gil-Yong Lee, Chan Lee, Gyu Hwan Park, Jung-Hee Jang

**Affiliations:** ^1^Department of Pathology, College of Oriental Medicine, Daegu Haany University, Daegu 42158, Republic of Korea; ^2^Department of Pharmacology, School of Medicine, Keimyung University, Daegu 42601, Republic of Korea; ^3^College of Pharmacy, Kyungpook National University, Daegu 41566, Republic of Korea

## Abstract

Increasing evidence suggests that neurodegenerative disorders such as Alzheimer's disease (AD) are mediated via disruption of cholinergic neurons and enhanced oxidative stress. Therefore, attention has been focused on searching for antioxidant phytochemicals for the prevention and/or treatment of AD through their ability to fortify cholinergic function and antioxidant defense capacity. In this study, we have investigated the neuroprotective effect of *α*-pinene (APN) against learning and memory impairment induced by scopolamine (SCO, 1 mg/kg, i.p.), a muscarinic receptor antagonist in C57BL/6 mice. Administration of APN (10 mg/kg, i.p.) significantly improved SCO-induced cognitive dysfunction as assessed by Y-maze and passive avoidance tests. In Morris water-maze test, APN effectively shortened the mean escape latency to find the hidden platform during training days. To further elucidate the molecular mechanisms underlying the neuroprotective effect of APN, the expression of proteins involved in the acetylcholine metabolism and antioxidant system was examined. Particularly, APN treatment increased mRNA expression of choline acetyltransferase in the cortex and protein levels of antioxidant enzymes such as heme oxygenase-1 and manganese superoxide dismutase in the hippocampus via activation of NF-E2-related factor 2. These findings suggest the possible neuroprotective potentials of APN for the management of dementia with learning and memory loss.

## 1. Introduction

Alzheimer's disease (AD) is a progressive neurodegenerative disorder characterized by hyperphosphorylated microtubule-associated protein tau, neurofibrillary lesions composed of the *β*-amyloid peptide, aberrant oxidative and inflammatory processes, and disturbance in neurotransmission [1]. AD produces significant cognitive impairment that arises from damage to cholinergic neurons known to play a crucial role in learning and memory functions. Cholinergic deficit has been regarded as a marker of neurological pathology that is associated with memory dysfunction and consistently correlated with the severity of cognitive impairment in AD [2]. Therefore, the recuperation of cholinergic role remains a coherent marker for developmental programs targeting the remedy of AD symptoms.

Scopolamine (SCO) is a muscarinic acetylcholine receptor (mAChR) antagonist which disturbs learning and memory function in animals and humans similar to those conditions observed in the patients with AD [3]. SCO has been used in establishing experimental models to evaluate the effects of therapeutic potential and benefits of drug candidates in dementia. In addition, SCO is considered to elevate acetylcholinesterase (AChE) levels in the cortex and hippocampus and has been used to search for and evaluate antidementia drugs [4].

Prolongation of acetylcholine (ACh) release into the neuronal synaptic cleft has been targeted as a means of increasing cholinergic functions in AD, which can be achieved by blocking enzymatic hydrolysis of acetylcholine by AChE [5]. In particular, acetylcholinesterase inhibitors (AChEI) such as rivastigmine (Exelon®), galantamine (Reminyl®), and donepezil (Aricept®) and cholinergic agonists are approved pharmacological therapies for AD. However, AChEIs have limitations such as short half-lives and severe negative side effects including diarrhea, nausea, vomiting, and hepatotoxicity [6]. Therefore, complementary and alternative therapies utilizing nontoxic phytochemicals from medicinal plants and foods for the prevention and/or treatment of AD are highly desirable and promising.

*Thuja orientalis *Linn., which is widely distributed in Korea and China, has been traditionally used to treat diarrhea, gout, rheumatism, and chronic tracheitis [7]. Recently, laboratory studies have shown that the extracts from* Thuja orientalis *Linn. exhibit a variety of biological functions such as antioxidant, anti-inflammatory, and antielastase activities [8–10]. Furthermore, one of the active components, *α*-pinene (APN), has been reported to possess antioxidant, immunostimulant, and anti-inflammatory properties [11–15].

Besides disturbance of acetylcholine metabolism in memory loss, recent studies assert that neuropathology of AD is associated with oxidative and inflammatory processes [5]. Reactive oxidative species (ROS) are able to deficit cellular constituents and play an important role as a secondary messenger mediating inflammation [16]. Prior researches reported that oxidative stress is associated with memory dysfunction in the SCO-induced animal model of amnesia [17–21].

In this study, we have investigated the memory enhancing effect of APN on SCO-induced memory deficit in C57BL/6 mice using the Y-maze, Morris water-maze, and passive avoidance tests. Moreover, in order to elucidate the molecular mechanisms underlying the neuroprotective effect of APN, the expression of proteins involved in the acetylcholine metabolism and antioxidant enzymes including heme oxygenase-1 (HO-1) and superoxide dismutase (SOD) were additionally examined.

## 2. Materials and Methods

### 2.1. Chemicals and Reagents

APN, SCO, and other chemical reagents were obtained from Sigma-Aldrich (MO, USA). Anti- MnSOD, anti-CuZnSOD, anti-GAPDH, and anti-NF-E2-related factor 2 (Nrf2) antibodies were provided by Santa Cruz Biotechnology (TX, USA). Anti-HO-1 and anti-pphospho-Nrf2 (p-Nrf2) antibodies were supplied from Stressgen (MI, USA) and Epitomics (CA, USA), respectively.

### 2.2. Experimental Animals

Male C57BL/6 mice (20–25 g, 8 weeks old) were purchased from the Dae Han Bio-Link. Co., Ltd. (Chungcheongbuk-do, South Korea) and acclimated to the colony room for 3 days. The animals were maintained with constant temperature (21–23°C) and humidity (55–60%) under a 12 h light-dark cycle (light on 7:00–19:00). Mice were allowed free access to food and fresh water during environmental adaptation and behavior test periods. The experimental procedure was conducted in accordance with the Care and Use of Laboratory Animals of the Daegu Haany University and Keimyung University.

### 2.3. Y-Maze Test

The Y-maze is a three-arm horizontal maze with an angle of 120 degrees, which were of 28 cm length, 6 cm width, and 18 cm height. The maze floor and walls were constructed with white polyvinyl plastic. Mice were initially placed in one arm, and then the sequence and number of arm entries were monitored for an 8-min period. An actual alternation was defined when a mouse entered into all three arms on consecutive choices (i.e., ABC, BCA, or CAB, but not CAC, BAB, or ABA). The spontaneous alternation (%) was derived from the total number of alternations divided by the total number of arm entries minus two, which was multiplied by 100 as shown in the following equation: % Alternation = [(Number of alternations)/(Total number of arm entries − 2)] × 100. The number of arm entries also served as an indicator for movement and locomotor activity.

### 2.4. Morris Water-Maze Test

The Morris water maze was conducted in a circular pool (120 cm in diameter and 45 cm in height) with a featureless inner surface. The water-maze tank was placed with four external visual cues and filled with water containing a nontoxic white color. The temperature of the water was maintained by 23–25°C. A white platform (10 cm in diameter and 30 cm in height) was placed in one of the quadrants with equal area and submerged 2 cm below the water surface. During each trial session, the escape latency time spent to find the hidden platform was monitored by a video tracking system (Ethovision System, Noldus, Wageningen, Netherlands). During the four subsequent days of training the mice were given three trials per day with the submerged platform in the pool. When the mouse located on the plated platform, it was allowed to remain on it for an additional 10 sec. If the mouse did not find the hidden platform within 120 sec, the mouse was guided to the platform and permitted to remain on it for an additional 10 sec. On the last day, the hidden platform was removed from the water-maze tank and probe test was performed. Mice were allowed to swim for 90 sec and the staying time in the maze quadrant where the platform had previously been located was recorded.

### 2.5. Passive Avoidance Test

The step-through passive avoidance test apparatus (Gemini Avoidance System, San Diego, USA) consisted of illuminated (25 cm × 20 cm × 20 cm) and nonilluminated chambers separated by a guillotine door (5 cm × 5 cm). The floor of illuminated and nonilluminated compartments was composed of 2 mm stainless steel rods spaced 6 mm apart. During acquisition test session, mice were gently placed in the illuminated chamber and 20 sec later the guillotine door between the two chambers was opened. When mice entered the nonilluminated chamber, the door was automatically closed and electrical foot shock (1 mA, 5 sec) was delivered through the stainless steel rods. After 24 h, during the retention trial session, the mice were placed again in the illuminated chamber. Then the step-through latency time to enter the nonilluminated chamber was measured. If the mouse did not enter nonilluminated chamber within 300 sec, the retention trial was ended and the step-through latency time was scored 300 sec as the upper limit.

#### 2.5.1. Reverse Transcription-Polymerase Chain Reaction (RT-PCR)

Total RNA was extracted from cortex of C57BL/6 mouse by TRI reagent (Molecular Research Center, OH, USA) according to the manufacturer's manual instruction. The sequences of primers were used in the polymerase chain reaction (PCR) using synthetic primers specific to choline acetyltransferase (ChAT), acetylcholine esterase (AChE), muscarinic acetylcholine receptors (M_1_ mAChR and M_2_ mAChR), and glyceraldehyde-3-phosphate dehydrogenase (GAPDH) as follows:

  ChAT
    5′-AGG GTG ATC TGT TCAS CTC AG-3′ (sense)
    5′-TCT TGT TGC CTG TCA TCA TA-3′ (antisense)
  AChE
    5′-AGA AAA TAT TGC AGC CTT TG-3′ (sense)
    5′-CTG CAG GTC TTG AAA ATC TC-3′ (antisense)
  M_1_ mAChR
    5′-CAG AAG TGG TGA TCA AGA TGC C-3′ (sense)
    5′-GAG CTT TTG GGA GGC TGC TT-3′ (antisense)
  M_2_ mAChR
    5′-TGC TGT GGC CTC CAA TAT GA-3′ (sense)
    5′-TGA CCC GAC GAC CCA ACT-3′ (antisense)
  GAPDH
    5′-GCC AAG GTC ATC CAT GAC AAC-3′ (sense)
    5′-AGT GTA GCC CAG GAT GCC CTT-3′ (antisense)

The specific region of a DNA strand was amplified by PCR mixture containing Taq polymerase (SolGent, Daejeon, Korea) under the following condition: denaturation step for 5 min at 95°C, then annealing step of 40 cycles for 60 sec at 51°C (ChAT), at 51°C (AChE), at 55°C (M_1_ mAChR), at 55°C (M_2_ mAChR), or at 57°C (GAPDH), and subsequent elongation step for 60 sec at 72°C. The amplified PCR products were analyzed by gel electrophoresis using 1.5% agarose gel prestained with ethidium bromide. Size separation images of the PCR products were evaluated by using QuantityOne Software of Image analysis Gel Doc XR System (Bio-Rad, CA, USA).

### 2.6. Western Blot Analysis

The hippocampus of C57BL/6 mouse were homogenized and incubated with RIPA buffer containing 50 mM Tris-HCl, pH 8.0, 1 mM EDTA, 10 mM NaF, 1 mM Na3VO4, 150 mM NaCl, 1% Triton X-100, and one complete protease inhibitor cocktail tablet (Roche diagnostics, IN, USA) on ice for 20 min. After incubation, the homogenates were centrifuged at 14,000*g* for 30 min at 4°C and concentration of protein was determined by using bicinchoninic acid (BCA) protein assay kit (Pierce, IL, USA). The equal amount of protein samples (30 *μ*g) were separated on 10–12% polyacrylamide gels and transferred to polyvinylidene difluoride (PVDF) membrane (Pall., MI, USA) at 300 mA for 3 hr. After transferring, the membranes were blocked with 5% nonfat dried milk in phosphate-buffered saline (PBS) with 0.01% Tween-20 (PBST) for 1 h at room temperature. The membranes were further incubated with primary antibodies at 4°C overnight. After three times washing with PBST, the membranes were reacted with horseradish peroxidase- (HRP-)conjugated anti-rabbit secondary antibody (Zymed, CA, USA) in 3% nonfat dried milk for 1 h at room temperature. The membranes were visualized with the enhanced chemiluminescence (ECL) Western blotting detection reagent (Amersharm Bioscience, NJ, USA) and images of specific target proteins were evaluated by using ImageQuant LAS 4000 Multi Gauge software (Fujifilm, Tokyo, Japan).

### 2.7. Statistical Analysis

The data were expressed as means ± SEM and statistical analysis for multiple comparisons was conducted by using one-way ANOVA followed by the Tukey test for a series of behavior tests with SPSS software (PASW Statistics 18.0 KO for windows). The data values were defined to be statistically significant at *p* < 0.05.

## 3. Results

### 3.1. Effect of APN on Memory Impairment Induced by SCO in Y-Maze Test

**Figure 1:**
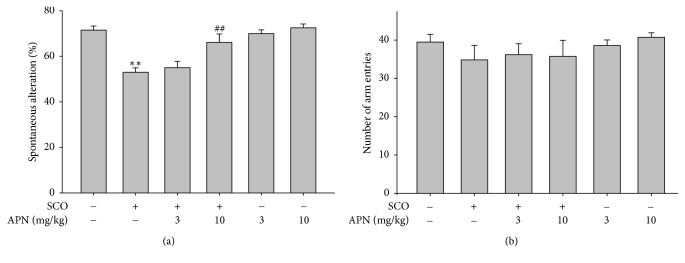
Memory enhancing effect of APN in Y-maze test. One hour before the test, mice were treated with vehicle or APN (3 or 10 mg/kg, i.p.) and, 30 min later, mice were injected with vehicle or SCO (1 mg/kg, i.p.). (a) Effects of APN on the SCO-induced spontaneous alternation. (b) Total number of arm entries in 8-min trials of Y-maze test. Data are represented as mean ± SEM (*n* = 7). Significant difference between the groups: ^*∗∗*^*p* < 0.05, vehicle-treated control versus SCO alone group; ^##^*p* < 0.01, SCO alone group versus APN-treated group in combination with SCO.

The SCO-induced amnesia group exhibited significantly reduced spatial recognition memory compared with sham control group in Y-maze test (Figure 1(a)). However, SCO-decreased recognition memory performance was effectively restored by intraperitoneal administration of APN (10 mg/kg, i.p.) (Figure 1(a)). The APN-treated group showed a higher spontaneous alternation score as compared with SCO-injected amnesic group. However, under the same experimental condition, the total number of arm entries was not much different among the experimental groups (Figure 1(b)).

### 3.2. Effect of APN on Memory Loss Induced by SCO in Morris Water-Maze Test

**Figure 2:**
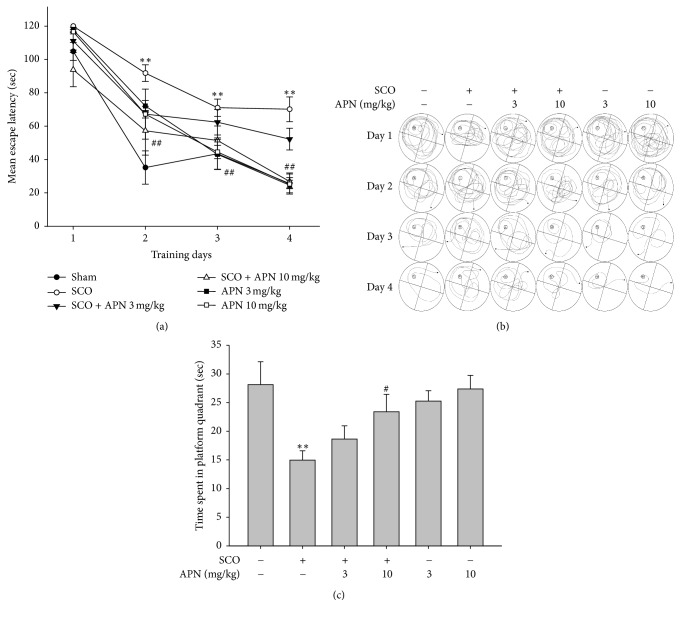
Protective effect of APN on the learning and memory deficit induced by SCO in Morris water-maze test. One hour before the test, mice were treated with vehicle or APN (3 or 10 mg/kg, i.p.) and then after 30 min injected with vehicle or SCO (1 mg/kg, i.p.). (a) Effect of APN on the SCO-induced spatial memory impairment was examined by mean escape latency. (b) The representative water-maze paths of each group were indicated during four training days. (c) On the last day of training trial, probe test was conducted in which the platform was removed from the pool and mice were allowed to swim and search it for 90 sec. Data are shown as mean ± SEM (*n* = 7). Significant difference between the groups: ^*∗∗*^*p* < 0.01, vehicle-treated control versus SCO alone group; ^#^*p* < 0.05 and ^##^*p* < 0.01, SCO alone group versus APN-treated group in combination with SCO.

We have conducted Morris water-maze test to confirm the memory enhancing effect of APN in C57/BL6 mice. During four consecutive training days, the mice in the sham group quickly found the location of the hidden platform than the SCO-induced amnesia group (Figure 2(a)). The SCO-injected group exhibited significantly delayed mean escape latency time compared with the sham control group from days 2 to 4. The group treated with APN (10 mg/kg, i.p.) significantly shortened the mean escape latency from days 2 and 4 (Figure 2(a)). At a dose of 10 mg/kg, the efficacy of APN was comparable to the sham group. As the higher doses of APN exhibited slightly lower efficacy than 10 mg/kg (data not shown), we have utilized up to 10 mg/kg as maximal effective dose of APN for subsequent behavior and molecular analysis. The representative pathways derived from each group during four training days were shown in Figure 2(b). In the probe trial, APN (10 mg/kg, i.p.)-treated group exhibited increased time spent in the quadrant where the platform has been placed (Figure 2(c)). Therefore, we demonstrated that APN enhanced spatial recognition in the Morris water-maze test.

### 3.3. Effect of APN on Cognitive Dysfunction Induced by SCO in the Passive Avoidance Test

**Figure 3:**
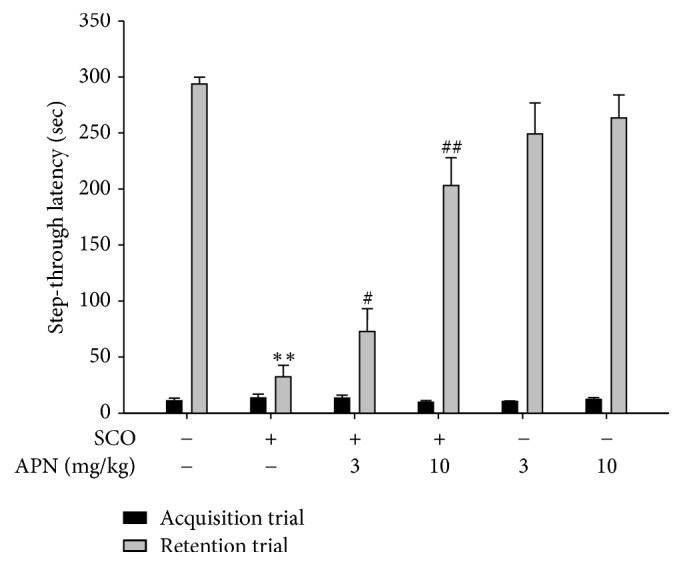
Protective effect of APN on the learning and memory deficit induced by SCO in passive avoidance test. During acquisition trial, one hour before this test, mice were treated with APN (3 or 10 mg/kg, i.p.) and, 30 min later, mice were injected with vehicle or SCO (1 mg/kg, i.p.). Effect of APN on the SCO-induced learning and memory deficit was monitored based on the step-through latency. Data are presented as mean ± SEM (*n* = 7). Significant difference between the groups: ^*∗∗*^*p* < 0.01, vehicle-treated control versus SCO alone group; ^#^*p* < 0.05 and ^##^*p* < 0.01, SCO alone group versus APN-treated group in combination with SCO.

During the acquisition trial of the step-through passive avoidance test, there were no significant differences among groups (Figure 3). However, during the retention trial which was performed 24 h after the acquisition trial, SCO-treated mice showed a significantly lower latency compared with sham control group (Figure 3). The decreased latency time indicates impaired retention of memory in the step-through type passive avoidance test. The SCO-induced memory impairment was effectively revered by administration of APN (10 mg/kg, i.p.) (Figure 3).

### 3.4. Effect of APN on the Expression of Enzymes and Receptors Involved in Acetylcholine Metabolism

**Figure 4:**
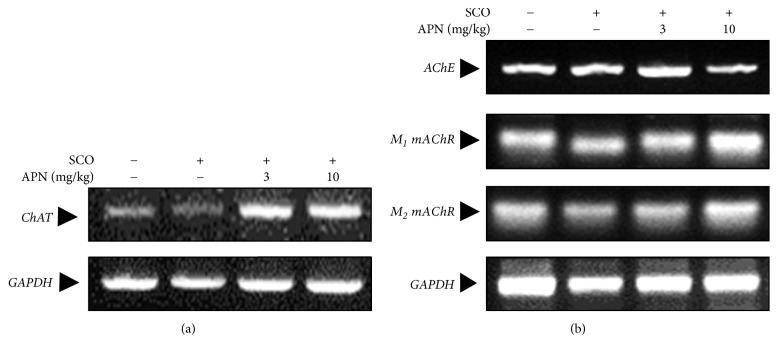
Effect of APN on the mRNA expression of enzymes and receptors related to acetylcholine. In the cortex of mice treated with SCO in the presence or absence of APN, mRNA levels of enzymes and receptors related to acetylcholine neurotransmission system such as (a) ChAT, (b) AChE, M_1_ mAChR, and M_2_ mAChR were determined by RT-PCR. GAPDH levels were compared to verify the equal amount of mRNA loading.

To elucidate the possible neuroprotective molecular mechanisms of APN, the mRNA levels of enzymes involved in the acetylcholine metabolism and acetylcholine receptors were assessed by RT-PCR. In the cortex, SCO slightly decreased the mRNA expression of ChAT which is to join acetyl-CoA to choline resulting in the formation of the acetylcholine (Figure 4(a)). Conversely, intraperitoneal administration of APN markedly elevated mRNA levels of ChAT (Figure 4(a)). Under the same experimental condition, the mRNA expression of AChE, which degrades the neurotransmitter ACh, and muscarinic acetylcholine receptors (M_1_ mAChR and M_2_ mAChR) was not significantly altered by SCO treatment in the presence or absence of APN (Figure 4(b)).

### 3.5. Effect of APN on the Expression of Antioxidant Enzymes

**Figure 5:**
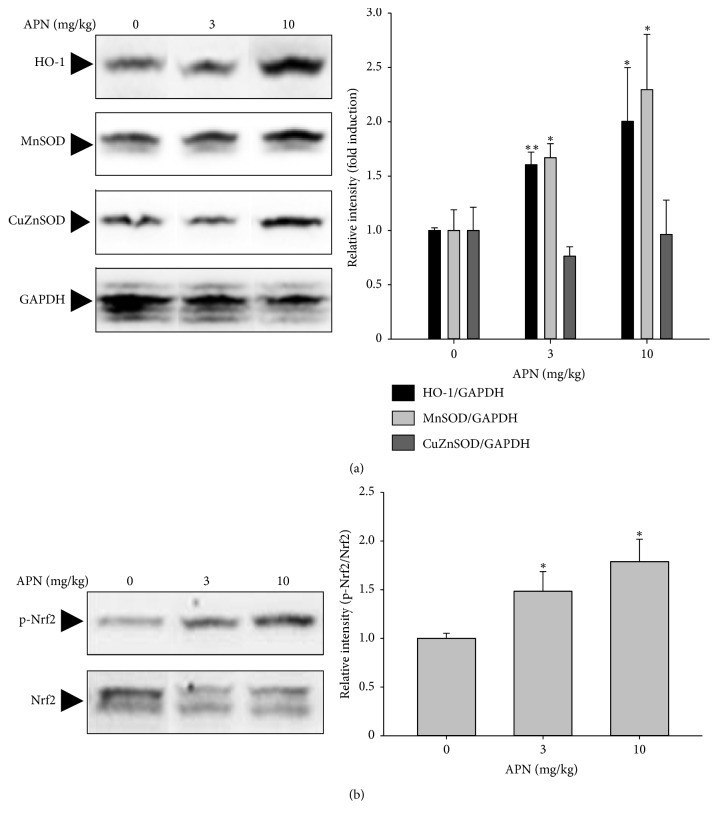
Effect of APN on the protein expression of antioxidant enzymes. (a) In the hippocampus of mice treated with SCO in the presence or absence of APN, protein levels of antioxidant enzymes such as HO-1, MnSOD, and CuZnSOD were determined by Western blot analysis. GAPDH levels were compared to normalize the equal amount of protein loading. (b) The protein expression of p-Nrf2 and Nrf2 was examined by Western blot analysis. Quantitative data of Western blot analysis was provided in the right panels. Significant difference between the groups: ^*∗*^*p* < 0.05 and ^*∗∗*^*p* < 0.01, vehicle-treated control versus APN-treated group.

To further verify the underlying molecular mechanisms of APN against SCO-induced learning and memory impairment, the protein expression of antioxidant enzymes was measured by Western blot analysis. In the hippocampus, APN effectively increased protein levels of HO-1 and MnSOD (Figure 5(a)). Under the same experimental condition, the expression of the aforementioned antioxidant enzymes in the cortex was not much altered by administration of APN (data not shown). Furthermore, in the hippocampus, treatment of C57BL/6 mice with APN led to an activation of a redox-sensitive transcription factor Nrf2 via phosphorylation (Figure 5(b)), which may regulate the subsequent expression of antioxidant proteins.

## 4. Discussion

In this study, we have evaluated the effect of APN, one of the major compounds in* Thuja orientalis *Linn., to potentially prevent and treat learning and memory deficits in an animal model of amnesia. We have examined whether APN attenuates learning and memory impairments induced by SCO (1 mg/kg, i.p.), a muscarinic acetylcholine receptor antagonist, by conducting Y-maze, Morris water-maze, and passive avoidance tests. SCO interferes with learning and memory functions and subsequently causes impairment of learning acquisition and short-term as well as long-term memories. Moreover, cholinergic neurons in the CNS are involved in mediating reference (long-term) as well as working (short-term) memories of both humans and animals [22]. Therefore, disruption of cholinergic neurotransmission system plays a critical role in the early stage of AD [23].

The spontaneous alternation score in the Y-maze test is an indication of working memory. SCO-induced reduction of spontaneous alternation score was increased by APN (10 mg/kg, i.p.) administration. The passive avoidance test is used as a useful tool for the estimation of working as well as reference memories. APN (10 mg/kg, i.p.) prolonged the step-through latency time and mitigated the memory impairments induced by SCO. The results suggest that APN may improve memory by rescuing the acetylcholine system from deficit caused by SCO.

The Morris water-maze test evaluates reference and spatial memories and detects changes in the central cholinergic system. The APN (10 mg/kg) shortened this escape latency time on days 2 to 4. At the probe trial session, APN (3 mg/kg and 10 mg/kg, i.p.) dose-dependently increased the time spent within the target quadrant. The simultaneous analysis for a distinction between working and reference memories is well established through the Morris water-maze test. The reduction in escape latency from day to day in the first trial represents reference memory, while that from first trial to second trial indicates working memory [24, 25]. Considering the escape latency time markedly reduced during the training trials, it seems likely that APN improves long-term and spatial memories in SCO-induced amnesic mice.

To further assess the neuroprotective mechanisms of APN, we have conducted a series of molecular analysis using cortical as well as hippocampal tissues. In both humans and animals these two brain areas particularly medial prefrontal cortex and hippocampus and their communications have been reported to play an essential role in encoding and retrieval of diverse memory processes [26]. In the cortex of SCO-induced amnesic animals, the mRNA levels of enzymes involved in the acetylcholine metabolism and muscarinic M_1_ and M_2_ AChRs were analyzed by RT-PCR. SCO lightly diminished the mRNA expression of ChAT in the cortical region. Reversely, intraperitoneal administration of APN resulted in a dramatic increase in the mRNA levels of ChAT. However, the mRNA expression of AChE which reduces the availability of ACh in the synaptic cleft was not changed by SCO treatment in the presence or absence of APN.

A number of studies have provided experimental evidence on the cholinergic hypothesis of learning, memory, and cognition [27]. The brain lesions of cholinergic nuclei or acetylcholine receptors (postsynaptic muscarinic M_1_ receptors and presynaptic muscarinic M_2_ receptors) and use of cholinergic antagonists are often related to cause cognitive dysfunctions similar to those observed in dementia [28–30]. Besides cholinergic hypothesis in learning and memory, many clinical and experimental researches reported that oxidative stress is involved in the pathological characteristics of neurodegenerative disorders including AD [31]. The activities and expression of antioxidant enzymes have been demonstrated to be altered in the pathology of neurodegenerative diseases [32]. According to recent studies, the dysfunction of learning, memory, and cognition induced by administration of SCO in animal models is associated with changes in the expression of antioxidant enzymes [33].

In this study, to confirm the molecular basis for the antioxidant mechanisms of APN, the protein levels of diverse antioxidant defenses enzymes were evaluated by Western blot analysis. In the hippocampus, treatment of C57BL/6 mice with APN led to an increased protein expression of HO-1 and MnSOD, which seemed to be mediated by activation of Nrf2 via phosphorylation. HO-1 is a rate-limiting enzyme that catalyzes the degradation of heme. This process produces biliverdin and carbon monoxide, which can ameliorate oxidative stress-mediated neurodegenerative disorders [34, 35]. SOD is an enzyme catalyzing the dismutation of superoxide radical into molecular oxygen or hydrogen peroxide (H_2_O_2_). Three isoforms exist in human such as cytoplasmic SOD1 (CuZmSOD), extracellular SOD3 (CuZnSOD), and mitochondrial SOD2 (MnSOD). In other studies APN has been reported to protect U373-MG [36] and PC12 [37] cells from H_2_O_2_-induced cytotoxicity as well as oxidative stress by increasing the activity and protein expression of endogenous antioxidant enzymes such as SOD, HO-1, glutathione peroxidase (GPx), glutathione reductase (GR), and catalase (CAT).

Nrf2 is a redox-sensitive transcription factor which is induced in the brain following toxic and/or subtoxic levels of stress and mediates induction of antioxidant and phase II detoxification enzymes such as HO-1, SOD, *γ*-glutamylcysteine ligase (GCL), glutathione S-transferase (GST), GPx, NAD(P)H:quinone oxidoreductase 1 (NQO1), and CAT [38]. Nrf2 has been reported to play crucial roles in neuroprotection in acute CNS injuries as well as diverse neurodegenerative disorders [38]. In C6 cells SCO treatment decreased the protein levels of Nrf2 and subsequent expression of downstream target genes including SDO, GPx, and CAT, which ultimately led apoptotic cell death via oxidative stress [39]. Conversely, in another study compound K derived from red ginseng exerted memory enhancing effect against SCO-induced amnesia via induction of Nrf2 as the protective effect of compound K was abolished in Nrf2 knockout mice [40].

In conclusion, present results demonstrate that APN exhibits memory enhancing activity and regulates the expression of proteins related to synthesis of acetylcholine and antioxidant defense system. Therefore, APN might be one of the useful natural agents of cure and/or prevention for amnesia and neurodegenerative diseases with learning, memory, and cognitive dysfunctions.
